# Comparable fracture risk between orexin and melatonin receptor agonists: integrating active-comparator and population-based evidence

**DOI:** 10.1007/s11657-025-01653-x

**Published:** 2025-12-22

**Authors:** Kei Muroi, Takashi Kanbayashi, Masashi Yanagisawa, Shun Nakajima

**Affiliations:** 1 International Institute for Integrative Sleep Medicine (WPI-IIIS), Tsukuba Institute for Advanced Research (TIAR), University of Tsukuba, Tsukuba, Ibaraki Japan; 2 Ibaraki Prefectural Medical Center of Psychiatry, Kasama, Ibaraki Japan; 3 Department of Molecular Genetics, University of Texas Southwestern Medical Center, Dallas, TX USA; 4 Institute of Medicine, University of Tsukuba, Tsukuba, Japan

**To the Editor,**

Tange and colleagues provide important active-comparator evidence on fracture risk with novel hypnotics [1]. Using Japanese claims data with a new-user design, they found no significant difference in fragility fracture risk between ramelteon (melatonin receptor agonist; MRA) and orexin receptor antagonists (ORAs) (adjusted hazard ratio [aHR] 1.07, 95% CI 0.99–1.17), with consistent findings across fracture types and age groups. This head-to-head comparison provides crucial clinical context for interpreting class-level fracture risk (Table 1).

**Table 1:** Comparison of fracture risk estimates: active-comparator vs. non-use comparisons

Study	Comparison	Outcome	Adjusted HR (95% CI)
Tange et al. [1]	MRA vs. ORA	Fragility fracture	1.07 (0.99–1.17)
Shibata et al. [2]	ORA vs. non-use	Hip fracture	3.09 (3.03–3.16)
Shibata et al. [2]	MRA vs. non-use	Hip fracture	2.45 (2.38–2.52)

*MRA* melatonin receptor agonist, *ORA* orexin receptor antagonist, *HR* hazard ratio, *CI* confidence interval

Viewed alongside recent population-based data, a clearer picture may emerge. Shibata et al. reported elevated hip fracture risk versus non-use for both ORAs (aHR 3.09, 95% CI 3.03–3.16) and MRAs (aHR 2.45, 95% CI 2.38–2.52) [2]. Sensitivity analysis using E-values indicates that unmeasured confounders would require associations of at least 5.6 (ORA) or 4.3 (MRA) with both exposure and outcome to explain away observed effects [3]. However, the E-value represents composite unmeasured confounding conditional on measured covariates [4], and multiple factors (e.g., insomnia severity, comorbidities, functional status) may collectively influence these associations. While these E-values suggest substantial confounding would be needed, they cannot conclusively demonstrate causality. Together with Tange et al. finding no difference between classes, these results suggest that both novel hypnotic classes may carry comparable fracture risk in routine clinical practice. 

Similar fracture risks despite different mechanisms suggest pharmacokinetic properties may contribute to risk across both classes. Ramelteon’s active metabolite (M-II) exhibits prolonged activity with substantially higher systemic exposure than the parent compound, while ORAs have long elimination half-lives causing residual next-day effects. Agent-specific analyses are warranted. 

These findings have important limitations. Shibata et al.’s study may reflect residual confounding, as it did not fully adjust for insomnia severity, comorbidities, and comedications [2]. Unexpected protective associations (female sex, anxiolytics, antihistamines) differ from previous reports, suggesting unmeasured confounding. Administrative claims data may not capture all relevant variables. These limitations highlight the value of integrating multiple evidence sources, including Tange et al.’s active-comparator design. 

These findings should be interpreted alongside previous evidence. While one meta-analysis found no significant association between ORAs and falls/fractures [5], this differs from real-world cohorts, likely reflecting study design and patient population differences. These findings have important clinical implications, particularly given high hypnotic prescribing rates in Japan. Cognitive behavioral therapy for insomnia (CBT-I) has demonstrated efficacy in older adults and should be first-line treatment [6]. Clinicians should exercise caution when initiating pharmacotherapy, as electroencephalography studies reveal discrepancies between subjective complaints and objective sleep in many insomnia patients [7]. Prescribing hypnotics to patients with objectively adequate sleep may increase unnecessary fracture risk [8]. Given the available evidence and its limitations, we recommend: (1) class-level caution with both ORAs and MRAs in high-risk patients, (2) prioritizing CBT-I, (3) considering objective sleep assessment, (4) avoiding long-term use, and (5) regular fracture risk reassessment.
